# Assessing the Functions of Non-Suicidal Self-Injury: Factor Analysis of Functional Assessment of Self-Mutilation among Adolescents

**Published:** 2019-07

**Authors:** Maryam Izadi-Mazidi, Hamid Yaghubi, Pavaneh Mohammadkhani, Hamidreza Hassanabadi

**Affiliations:** 1 Department of Clinical Psychology, School of Humanities, Shahed University, Tehran, Iran.; 2 Department of Clinical Psychology, University of Social Welfare and Rehabilitation Sciences, Tehran, Iran.; 3 Department of Educational Psychology, School of Psychology and Education, Kharazmi University, Tehran, Iran.

**Keywords:** *Adolescent*, *Assessment*, *Functional Assessment of Self-Mutilation (FASM)*, *NonSuicidal Self-Injury*

## Abstract

**Objective:** The aim of this cross sectional study was to assess the factor analysis of Functional Assessment of Self-Mutilation (FASM) among Iranian adolescents with non-suicidal self-injury.

**Method**: In this study, 646 high school students, with the mean age of 16.55 ± 0.7, were selected using a multistage cluster sampling method; they completed FASM and the demographic form. Data were analyzed using the descriptive statistics, chi-square (χ2), independent sample t test, MANOVA, and confirmatory factor analyses (CFA).

**Results**: Of the participants, 178 reported at least 1 episode of NSSI during the previous year. The mean age of the participants when they first harmed themselves was 14.64 (±1.71). Most of them reported to engage in NSSI impulsively (39.32%) and experienced little (31.5%) or moderate physical pain (31.5%) There were no significant differences between males and females in severity of NSSI, frequency of NSSI, thinking about NSSI prior to engaging in the act, and age of onset. The results of the confirmatory factor analysis supported the 4-factor model of NSSI functions suggested by Nock and Prinstine [Χ2/df = 1.84; RMSEA = 0.07; GFI = 0.82; AGFI = 0.77]. The most frequent function for engaging in NSSI was Automatic Negative Reinforcement.

**Conclusion**: Findings of this study supported the structural validity of the FASM; thus, this tool can be useful in treatment and research contexts as a measure of NSSI functions. Moreover, this study found that adolescents engage in non-suicidal self-injury because of 4 distinct reinforcement processes. The study findings have important implications for the assessment and treatment of NSSI.

Non-suicidal self-injury (NSSI) is deliberate, socially unacceptable destruction of one’s own bodily tissue without suicidal intent (1). Methods of NSSI include hitting, scratching, banging, interfering with wound healing, cutting, and burning (2).

Adolescents are the most vulnerable group for NSSI. In a meta-analysis, NSSI prevalence rate was found to be 17.2% among adolescents in nonclinical samples (3). The lifetime prevalence rate of NSSI has been reported to be 21.3% in Iranian university students, the majority of whom engaged in NSSI from adolescence (4). In a study by Peivastehgar (2013), 12% of Iranian adolecent girls reported one episode of NSSI during the last year and 4% reported two NSSI episodes or more (5). 

This prevalent behavior can result in serious physical consequences such as physical injury, infectious disease, medical complications, and death (6-9). Moreover, it leads to aversive feelings and emotions (e.g., shame, guilt, and anger), emotional distress (8, 9), and impairment in academic performance (6, 10). It is also a potent predictor of suicidal attempt (11-13). Therefore, understanding the fundamental features of etiology and underlying mechanisms of NSSI is necessary to develop methods to prevent NSSI.

During the past several decades, a broad range of theories have been proposed to address why people engage in NSSI. Early perspectives suggested that people harm themselves to end or elicit dissociation (14-16), create boundary between the self and others (17), control their sexual impulses (17,18), replace suicide (17,19), or externalize emotion to punish the self or protect others (14,15,18). 

These theories have not received strong empirical support; moreover, each of them fall short in addressing the multitude of reasons of engaging in NSSI (20).

Nock and Prinstein (2004) developed a Four-Function Model (FFM) which represented advancement from prior accounts of self-injury. 

Based on functional approaches, which are derived from the behavioral perspective, behaviors are largely controlled by immediate antecedents and consequences (i.e., events that immediately precede and follow them) (21). According to FFM, NSSI is maintained by 4 following processes : (1) automatic negative reinforcement (ANR; i.e., NSSI followed by a decrease or elimination of aversive emotions or cognitions); (2) automatic positive reinforcement (APR; i.e., NSSI followed by an increase or generation of positive feelings or cognitive states); (3) social negative reinforcement (SNR; i.e., NSSI followed by a decrease or elimination of aversive social events); and (4) social positive reinforcement (SPR; i.e., NSSI followed by an increase or generation of desired social events) (21-23). 

Chapman, Gratz, & Brown (24) and Klonsky (25) have also proposed theoretical models to address the reasons for engaging in NSSI, but they only focused on the role of emotion regulation in self-injury and placed less emphasis on social functions, whereas FFM integrates automatic and social reinforcement within a comprehensive account.

In some studies in the USA (21, 22 and 26) and Canada (27), four-factor model of NSSI was confirmed through confirmatory factor analysis (CFA). In these studies, the most widely supported function was ANR. In a research on a Swedish community sample, Zetterqvist, Lundh, Dahlstrom, and Svedin (28) found that a two-factor model (social reinforcement and automatic reinforcement) resulted in better fit. Among Chinese adolescents, You, Lin, and Leung (29) found support for a three-factor structure of functions: affect regulation, social influence, and social avoidance.

Cultural factors have an impact on NSSI, its risk factors, and gender differences (30). American psychiatry association also excluded the culturally sanctioned self-injury from DSM-5 Criteria for NSSI disorder (31). Nonetheless, study of the functions of NSSI has received little research attention in Iran. Thus, the present study aimed to assess the functions of NSSI among Iranian adolescents who engage in NSSI. Knowledge in this area has the potential to identify the most effective interventions for NSSI.

## Materials and Methods

***Participants***

This cross sectional study was conducted from April to August, 2018. The participants consisted of 646 students (352 boys and 294 girls), with an age range of 15 to 18 years, (mean age: 16.55 ± 0.7). They were selected from 12 schools in different localities of Tehran/Iran (north, sought, west, and east) by multistage cluster sampling method. Each participant was asked to complete the Persian version of Functional Assessment of Self-Mutilation (FASM), a questionnaire used to obtain demographic data and to screen trichotillomania, excoriation, and psychotic disorders.

Inclusion criteria were age range of 15-18 years and willingness to participate in the study. Exclusion criterion was the presence of trichotillomania, excoriation, or psychotic disorders.

The participants were informed of their right to leave the study at any time. All the personal information was kept confidential. The study was approved by the ethics committee of Shahed University (No: IR.SHAHED.REC.1397.001).

***Measures***

***Functional Assessment of Self-Mutilation (FASM)***

This instrument was designed by Lloyd et al (32) to assess frequency, functions, and other characteristics of self-mutilation behavior (SMB), including the degree of physical pain, amount of time they thought about engaging in SMB, and the use of alcohol or drugs during self-injury. FASM consists of two sections. The first section of the scale is a checklist of 11 self-injury behaviors (cutting the skin, burning the skin, self-biting, scratching the skin, inserting objects to the nail or skin, self-punching, picking at wound, and pulling hair, erasing the skin, self-tattooing). 

NSSI behaviors are classified into two types of moderate/severe (cutting, burning, erasing the skin, and self-tattooing) and mild (pulling hair, inserting objects under nails or skin, biting self, hitting self, picking at a wound, scratching skin, self-punching).

Those who endorsed at least one NSSI behavior were instructed to complete the second section which included 22-item questions about the reasons for self-injury. The items are rated on a 4-point Likert scale, ranging from 0 “never”, 1 “rarely”, 2 “some” to 3 “often”.

***The Persian Version of FASM***

 The English version of the FASM was translated into Farsi. Then, the translation accuracy was checked by three psychology professors who approved the face validity of the FASM. In the next step, the approved version was translated back into the original language by two translators (an English language specialist familiar with the psychology texts and a psychologist fluent in both English and Farsi). The mismatched cases were resolved after comparing the retranslated version with the original version of the FASM.

***Statistical Analysis***

Data were analyzed using the descriptive statistics, chi-square (χ2), independent sample t test, MANOVA, and confirmatory factor analyses (CFA). Confirmatory factor analysis was conducted to explore the factor structure of NSSI functions. Model fit was evaluated using multiple indices of fit, including relative/normed chi-square (χ2/df), root mean square error of approximation (RMSEA), goodness-of-fit index (GFI), adjusted goodness-of-fit index (AGFI), and Akaike information criterion (AIC).

Acceptance of models was based on the following criteria: χ2/df<2 (33), RMSEA < 0.08, GFI>80, AGFI>80(34). In AIC, lower indices indicate preferred models. Statistical analyses were done using SPSS version 16 and LISREL 8.8.

## Results

Demographic features of participants are presented in Table 1. Of the whole sample, 76 male (42.69%) and 102 female (57.30%) students reported at least one episode of NSSI during the last year.

***Descriptive Characteristics of NSSI***

Among the participants who engaged in NSSI, 69.2% endorsed one or two episodes of NSSI and 30.8% of them did NSSI repetitively (three times or more) during the last year. Also, 69.9% of the participants engaged in severe/ moderate NSSI (with or without mild form of NSSI), and 30.1% reported mild NSSI.

Among the individuals with NSSI, 66.4% reported cutting their skin (with or without other forms of NSSI), 13.7% reported just hitting themselves, 2.7% reported just pulling their hair out, 1.4% reported just bitting themselves, and 15.8% reported more than 1 form of NSSI, excluding cutting the skin. There was no report of self-burning.

There were no significant differences between individuals with and without NSSI in gender, ethnicity, and mother and father's education (all p > 0.05; Table 1). Also, there were significant differences between the two groups in educational major and residential area (P < 0.001)

Moreover, no significant differences were found between males and females in severity of NSSI (P = 0.09), frequency of NSSI (P = 0.1), thinking before doing the harm (P = 0.7), and age of onset (P = 0.1).

***Contextual Features of SMB***

Most participants did not contemplate before performing each incident and reported no pain during each incident (Table 2). The mean age of the participants when they first harmed themselves was 14. 64 (±1.71).

**Functions of NSSI**

The four-factor model suggested by Nock and Prinstein (21, 22), the three-factor model identified by

You, Lin, Leung (29), and two-factor in the study by Zetterqvist et al (28) were compared on goodness-of-fit. A summary of the CFA results for the three models are presented in Table 3.

Results of this study showed that four-factor model best represented the data and was preferred over the two-factor and three-factor models in this sample according to smaller Akaike information criterion (AIC) index (Table 3).

***Differences in Functions of NSSI***

Scores for items belonging to each factor were summed to form A-NR, A-PR, S-NR, and S-PR functions scales. To compare endorsement of four functions, the scores were prorated by dividing the scales' scores by the number of subscales on each scale (two for A-NR, three for A-PR, four for S-NR, and 12 for S-PR).

A-NR (prorated; M = 0.95, SD = 0.84) and A-PR (prorated; M = 0.81, SD = 0.76), respectively, were more endorsed than SNR (prorated; M = 0.38, SD = 0.52), and S-PR (prorated; M = 0.38, SD = 0.42).

A significant difference was observed between male and female students in ANR function, with higher scores in females (F = 4.05, P = 0.04). Differences in other functions were not statistically significant (all p > 0.05; Table 4).

## Discussion

The present study assessed the prevalence of NSSI in a community sample of Iranian adolescents and found it to be a prevalent condition. This behavior reported to begin at a relatively middle adolescence, 14.64 (±1.71) years. In a study conducted by Gholamrezaei et al (4), the mean age of onset of NSSI was 13.7 (± 5.15) years among Iranian university students. However, Nock and Prinstein (21) and Lloyd-Richardson et al (26) found that most participants began engaging in self-injury in early adolescence (12.8± 2.1 and 12·87 ±2.94, respectively). 

It seems that NSSI occurred at an older age in Iranian adolescents compared to American adolescents. In a study by Zetterqvist et al (28), the mean debut age for NSSI was 13.9 (±1.7) years among Swedish adolescents.

In the present study, NSSI was more prevalent in south and east regions of Tehran, which may be due to the lower socioeconomic status of people in these regions which exposed them to psychological problems. 

 There were no significant differences between male and female individuals in the 12-month prevalence of NSSI, which confirmed the results of earlier studies (21, 26, 35-38). By contrast, some research (28, 29, 39 and 40) found that females were significantly more likely than males to engage in NSSI. Tang et al (41) and Izutsu et al (42) reported more prevalence of NSSI in males compared to females.

In the present study, no significant difference was found between male and female participants in severity and frequency of NSSI, thinking about NSSI prior to engaging in the act, and the age of onset. In agreement with these results, Nock and Prinstein (21) did not report a significant gender difference for frequency, methods, or age of onset of NSSI. Also, in a study by Lloyd-Richardson et al (26), no difference was found in severity of NSSI based on gender. However, Zetterqvistet al (28) found that a greater proportion of girls (compared to boys) conducted more than 5 episodes NSSI during the last year. The difference was statistically significant.

The differences in results among studies which have been done on various populations could be partially explained by the different samples across the studies or cultural differences.

***The Functions of NSSI***

**Figure 1:**
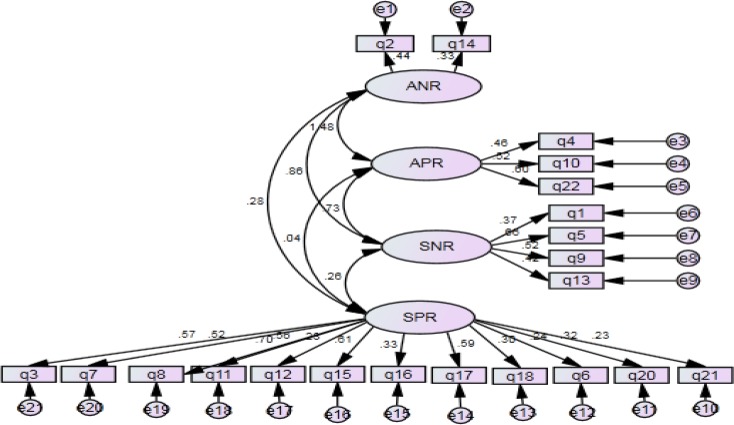
Factor Model Fitted for FASM Data among Adolescents

In the present study, a confirmatory factor analysis (CFA) was conducted on reported functions of NSSI to validate Nock and Prinstein’s four-functional model (FFM) on Iranian high school students (Figure 1). Also, a four-factor model resulted in a better fit compared to the three-factor model (29) and two-factor model (28)(Table3), supporting the four distinct reinforcement processes (ANR, APR, SNR, and SPR) that cause NSSI (21, 22). Consistent with previous research (21, 25, 43, 28), emotion regulation (primarily ANR) was more endorsed than regulation of social environment. 

These findings provide clear directions for clinical works. The consideration of the function of NSSI can guide one’s clinical conceptualization. The support for the existence of four functions of NSSI suggests that functionally relevant strategies may be warranted for NSSI treatment. Clinicians should tailor interventions accordingly and use different treatment approaches depending on the function of NSSI.

The finding that emotion regulation is a prevalent reason for engaging in NSSI highlights the importance of addressing this issue in treatment of NSSI; and interventions that focus on emotion regulation would be most relevant (20). Social functions should also be addressed although they are not as frequent as emotion regulation. However, the strategies that promote effective interpersonal communication and emotional expression skills and problem-solving may be appropriate (20). The findings could also help clinicians to identify individuals at risk of NSSI and help them by taking practical preventive measures. 

In the present study, female students scored higher in ANR function compared to males, which may be due to differential socialization patterns. Females are more likely to direct their feelings inward (29). Moreover, females experience physical and sexual abuse more than males (35). Cognitive and emotion dysregulation could mediate the relation between these experiences and self-injury (43).

Finally, the findings showed that FASM may be useful in research and treatment contexts when a thorough assessment of NSSI functions is needed.

**Table 1:** Demographic Features of Participants and Prevalence of Self-Injury According to Demographic Variables among Adolescents

**Demographic** **variable**		**Frequency N (%)**			**P-value**
		**Total**	**Self-injurers**	**Non self-injurers**	
**Gender**	Male	294(45.51)	76(42.69)	218(46.58)	0.2
	Female	352(54.48)	102(57.30)	250(53.41)	
**Ethnicity**	Fars	377 (58.35)	100(56.1)	277 (59.18)	0.2
	Turk	128(19.81)	41(23.03)	87(18.58)	
	Lor, Kord and Arab	69(10.68)	23(12.92)	46(9.82)	
	Other	25(3.89)	4(2.24)	21(4.48)	
**Educational ** **Major**	Mathematics	198(30.1)	37(20.78)	161(34.4)	<0.001
	Human Sciences	253(40.0)	92(51.68)	161(34.4)	
	*Experimental* Sciences	181(28.6)	41(23.03)	140(29.91)	
**Mother** **Education**	High school and Lower	164(28.4)	50(31.25)	114(27.27)	0.5
	Diploma and Associate Degree	232(40.1)	57(35.62)	175(41.86)	
	Bachelor	125(21.6)	37(23.12)	88(21.05)	
	Post graduate	57(9.9)	16(10)	41()	
**Father ** **Education**	High school and Lower	154(26.1)	52(32.09)	102(23.88)	0.2
	Diploma and Associate Degree	237(40.2)	59(36.41)	178(41.68)	
	Bachelor	119(20.2)	29(17.9)	90(21.07)	
	Post graduate	79(13.4)	22(13.58)	57(13.34)	
**Residential ** **area**	North	121(18.73)	28(15.73)	93(19.87)	P<0.001
	South	282(43.65)	96(53.93)	186(39.74)	
	East	128(19.81)	41(23.03)	87(18.58)	
	West	115(17.8)	13(7.3)	102(21.79)	

**Table 2:** Descriptive Statistics for Contextual Features of SMB among Adolescents

Variable	**N**	**%**
**Pain**		
No pain	39	21.9
Little pain	56	31.5
Moderate pain	56	31.5
Severe pain	11	6.2
**Contemplation**		
Not at all	70	39.32
A Little Bit	28	15.7
Somewhat	1	0.7
Very Much	14	9.6
Extremely	5	3.4

SMB: self-mutilative behavior

**Table 3:** Summary of the CFA Results of the 3 Models of FASM among Adolescents

Model	Χ^2^	Df	Χ^2^/df	GFI	AGFI	RMSEA	AIC
2-factor	438.36	188	2.33	0.78	0.73	0.09	524.36
3-factor	646.52	206	3.13	0.71	0.65	0.1	740.52
4-factor	337.61	183	1.84	0.82	0.77	0.07	433.61

**p < 0.01; *p < 0.05

CFA: confirmatory factor analysis

FASM: Functional Assessment of Self-Mutilation

**Table 4:** Gender Differences in Functions of NSSI among Adolescents

	**gender**	**Prorated Mean**	**Std. Deviation**	**F**	**P-value**
**ANR**	Male	0.77	0.85	4.05	0.04
	Female	1.06	0.82		
**APR**	Male	0.79	0.73	0.06	0.8
	Female	0.83	0.77		
**SNR**	Male	0.35	0.4	0.36	0.5
	Female	0.4	0.58		
**SPR**	Male	0.38	0.47	0.00	0.9
	Female	0.38	0.39		

NSS: Non-Suicidal Self-Injury

## Limitation

The findings of the study need to be interpreted in the context of some limitations. FASM is a self-reporting questionnaire; therefore, it is possible that participants were not completely honest in responding to the questions because NSSI and some of its functions are not socially desirable. However, the anonymous format and assurance of confidentiality increased the probability of truthful answers. Also, retrospective self-reports have the limitation of memory bias. Moreover, the absence of an external criterion which could judge the validity of the self-report measures was another limitation. Finally, the cross sectional design of the study precluded any conclusions concerning causal relationships.

## Conclusion

Non-suicidal self-injury is a prevalent behavior in Iranian adolescents and occurs due to intrapersonal and interpersonal problems. The most frequent reason for engaging in NSSI was a decrease or elimination of aversive emotions or cognitions (ie, ANR function). This finding has the potential to identify the most efficacious psychological interventions for NSSI. FASM is a comprehensive measure of NSSI functions that may be useful in research and treatment contexts.
